# Characterizing the Drug-Release Enhancement Effect of Surfactants on Megestrol-Acetate-Loaded Granules

**DOI:** 10.3390/ph15020113

**Published:** 2022-01-18

**Authors:** Gábor Katona, Bence Sipos, Rita Ambrus, Ildikó Csóka, Piroska Szabó-Révész

**Affiliations:** Faculty of Pharmacy, Institute of Pharmaceutical Technology and Regulatory Affairs, University of Szeged, H-6720 Szeged, Hungary; sipos.bence@szte.hu (B.S.); ambrus.rita@szte.hu (R.A.); csoka.ildiko@szte.hu (I.C.); reveszpiroska@szte.hu (P.S.-R.)

**Keywords:** quality by design, surfactant, polymeric micelle, megestrol-acetate, drug release, melt technology

## Abstract

In this study, the effect of Cremophor^®^ RH 40 (CR 40) classic micelles and Soluplus^®^ (SP) polymeric micelles were investigated on a novel granule-type drug-delivery system containing megestrolacetate (MGA). Using a risk assessment-based approach on the formulation via melt technology resulted in the formation of these granules, presented as the dosage, with proper particle size and flow characteristics. Due to the application of a eutectic carrier base composition, gentle process conditions were reached, retaining the crystalline structure of the carrier system and allowing for the proper distribution of MGA in the granules. The increased water solubility (0.111 mg/mL to 2.154 mg/mL), and the decreased nano particle size (102.27 nm) with uniform distribution (polydispersity index of 0.259) and colloid stability (zeta potential of −12.99 mV) resulted in SP polymeric micelles prevailing over CR 40 micelles in this gastric dissolution study, performed in biorelevant fasted and fed state drug-release media. Mathematical characterization and kinetic model fitting supported the fast drug-release mechanism of polymeric micelles over micelles. The value-added polymeric micelle-containing formulation developed can be successfully administered perorally and the enhanced drug release offers the possibility of greater drug absorption in the gastrointestinal tract.

## 1. Introduction

Surface-active excipients have been widely used in the pharmaceutical industry and in research and development processes due to their advantageous effects on drug applicability, solubility, and permeability enhancement [1]. Classic surfactants are most commonly used to decrease the surface tension between oily and aqueous phases in emulsions or to stably suspend water-insoluble drugs. However, the main focus has become a specific group of surfactants, amphiphilic graft co-polymers, which are able to form nanoparticles in the form of polymeric micelles [2,3,4].

Cremophor^®^ RH 40 (CR 40) is a hydrogenated castor oil used as a nonionic solubilizer and emulsifying agent to solubilize hydrophobic active substances in aqueous and alcoholic dosage forms. They form “ex tempore” small (10–30 nm) classic micelles besides physically coating the particles to keep them suspended or solubilized [5]. The concentrations of CR 40 in oral formulations ranges from 2 to 600 and from 25 to 405 mg (FDA database for Inactive Ingredients in Approved Drug Products) [6]. Soluplus^®^ (SP) is a novel solubilizing amphiphilic graft copolymer (polyvinyl caprolactam-polyvinyl acetate-polyethylene glycol graft copolymer (PCL-PVAc-PEG) offering better solubility enhancement in combination with rapid drug release due to spontaneously micelle formation (~80 nm) above the critical micelle concentration (CMC). Based on the CMC values, it can be claimed that less is needed to offer the required surfactant effect on the drug particles (Table 1). Surfactants in general can cause cell degradation when used in excess amounts, but due to the low toxicity of both CR 40 and SP, they are safely applied for oral administration [7,8,9].

In this study, the suitability of these surfactants in the case of melt technology was investigated to develop a megestrol-acetate (MGA)-loaded granule formulation. Previously, a crystalline carrier composition composed of xylitol (XYL), mannitol (MAN), and polyethylene glycol 6000 (PEG 6000) was developed. The eutectic mixture of these sugar alcohols results in decreased melting temperature of pure MAN (169.5 °C to approx. 91 °C), which is preferential for performing melt granulation in gentle conditions (lower temperature). However, the melted mixture of XYL and MAN results in rapid solidification; therefore, it is necessary to apply PEG 6000 as a softener material, which has no influence on the recrystallization of the components but offers better processability [10].

MGA is generally used in the treatment of weight loss in cachectic patients who are older or who have HIV and for cancer. It is used in breast and endometrial cancer, and it can also be applied in birth control. MGA is a lipophilic active substance (logP = 3.2) with poor water solubility and poor emulsion forming properties. The therapeutic peroral dose is usually 160–400 mg due to its bad bioavailability, which results in excess side-effects. It is classified as a Biopharmaceutical Classification System (BCS) Class II drug, which means it has poor water solubility but good intestinal permeability. Via enhancing the water solubility and drug release, a highly effective, permeable concentration can be achieved in the gastrointestinal tract, which opens the possibility of lowering the dose [11,12,13].

The aim of this work was to develop a new type of water-soluble crystalline carrier system from a eutectic of two sugar alcohols (XYL and MAN) with PEG 6000 (XMP) containing MGA using melt technology. The effects of two solubilizing agents (CR 40 and SP) on melt technology, solubility, and in vitro drug release were investigated. In this comparison study, the aim was to emphasize the differences between the solubilizing agents (MGA-CR 40 and MGA-SP formulations), focusing on the advantageous properties of SP-based polymeric micelles, which could be an effective way to increase the bioavailability of MGA. The quality-by-design-based holistic and systematic experiment design and the in vitro characterization focused on the mathematical evaluation of different biorelevant drug-release medias.

## 2. Results

### 2.1. Quality-by-Design-Based Risk Assessment

**Figure 1:**
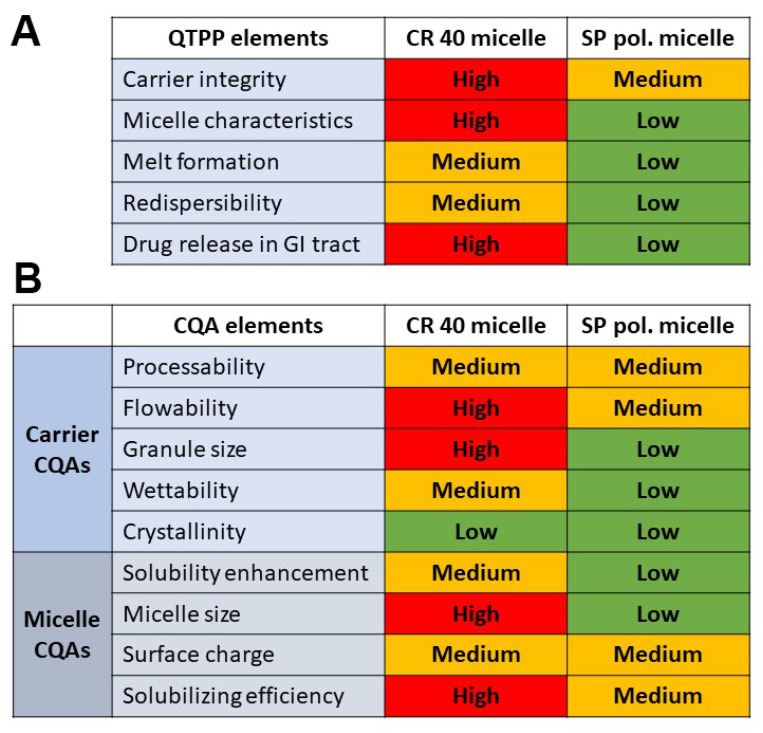
Comparative interdependence rating amongst CR 40 micelles and SP polymeric micelles: (**A**) the selected QTPP elements and (**B**) the selected carrier and micelle CQA elements.

The quality-by-design-driven risk assessment was performed to determine the risk factors related to the incorporation of the surfactants into the melt technology-produced MGA-loaded granules. A comparative analysis was performed where the selected Quality Target Product Profile (QTPP) and Critical Quality Attributes (CQAs) were evaluated by the effect of CR 40 micelles and SP polymeric micelles. The three-level scale (high–medium–low) was determined for each relation as can be seen in Figure 1.

When assigning the relation severity, the physicochemical properties of each surfactant were taken into consideration along with prior knowledge and experiments about these materials and technology. The QTPP elements stand for the general aim of the development process: carrier integrity—to achieve macro-sized particles and monodisperse distribution with uniform API content; micelle characteristics—to achieve nanosized micelles (CR 40) and polymeric micelles (SP) with monodisperse size distributions and adequate stability-enhancing surface-charge properties; melt formation—the surfactants should be incorporated homogenously into the melt of MGA and the carrier; re-dispersibility—the formulations should be easily re-dispersible in water, resulting in the self-assembly of (polymeric) micelles and an enhancement in drug release in the gastrointestinal tract. The CQA elements were divided in two to investigate the carrier-related and the carrier formulability-related CQA elements and the redispersed micelle characteristics as well.

**Figure 2:**
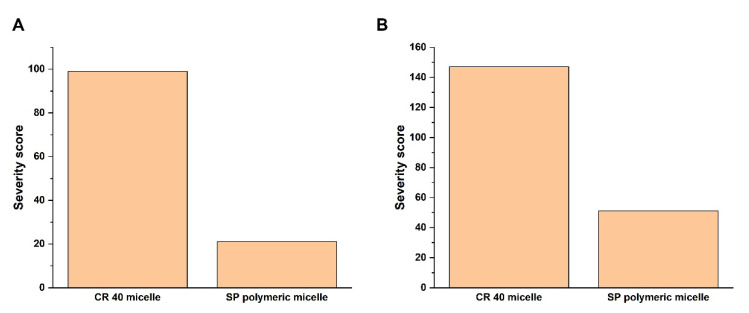
Probability rating as the calculated severity scores of the QTPP (**A**) and CQA (**B**) elements with a comparison of CR 40 micelles and SP polymeric micelles.

To quantify the relations based on interdependence and occurrence, a probability rating was performed using software. The results are depicted as Pareto column charts, which can be seen on Figure 2.

The software-calculated severity scores support that the SP polymeric micelles have a lower risk on the desired final product compared with the CR 40 micelles. This is due to the advantageous properties of the amphiphilic graft co-polymer-type SP, which for example has higher solubilizing efficiency and burst-type drug-release enhancing effects compared with other classic surfactants. Based on the selected CQAs along with the desired QTPP elements, structure, carrier, and micelle characterizations were further performed.

### 2.2. Characterization of MGA in the Carrier

#### 2.2.1. Thermoanalytical Measurements

Differential scanning calorimetry (DSC) and thermogravimetry (TGA) measurements were carried out to test the behavior of MGA and the formulations against temperature increase. The temperature interval was 25 to 240 °C in both measurement cases.

**Figure 3:**
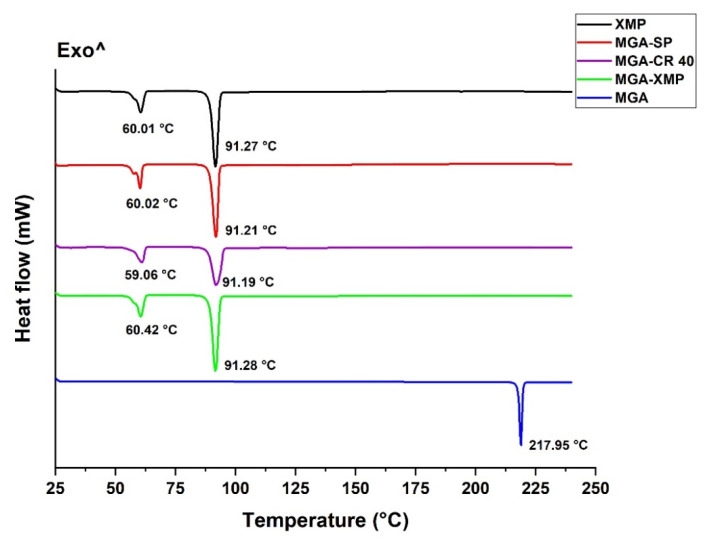
DSC curves of raw MGA, the carrier XMP, the surfactant-free MGA-containing formulation (MGA-XMP), and the CR 40 containing (MGA-CR 40) and SP containing (MGA-SP) granule formulations.

On the DSC thermogram of crystalline MGA, a characteristic melting point at 217.95 °C can be observed, which has disappeared in the thermograms of the surfactant-free and the surfactant-containing granule formulations (Figure 3). On these thermograms, besides the characteristic peaks of PEG 6000 and XYL-MAN (XMP), no other peaks can be observed that may arise from a crystalline fraction. This supports the successful melting and dispersing of MGA in the molten XMP system. The peak around 60 °C is assigned to the melting point of PEG 6000, and the peak around 91 °C is the eutectic melt of XYL-MAN. The effect of the surfactants on these characteristic peaks has been calculated from the integral value of these peaks normalized to the sample weight (Table 2). A slight decrease can be observed in the melting enthalpy absolute values of the MGA-CR 40 and MGA-SP formulations compared with MGA-XMP. The bigger difference is in the case of the MGA-SP formulation, whereas the initial −42.15 and −116.83 J/g enthalpy values increase to −32.61 and −86.18 J/g for the peaks of PEG 6000 and XYL-MAN, respectively. It has been demonstrated before that surfactant (and other type of polymers, lipids) can decrease the required heat enthalpy needed to break the bonds and allow the melting of mixtures; this theorem is supported by our results as well [14]. Based on this, it can be claimed that the addition of surfactants to the formulation is beneficial due to reasons besides the possible advanced pharmacokinetic effects. 

Thermogravimetric analysis (TGA) was performed to investigate the thermal stability of MGA and the granule formulations. The degradation was examined in the temperature interval of 25 to 240 °C. The weight loss was measured, which can be seen in Table 3, showing that the granule formulations and MGA are stable against the temperature increase. In case of the MGA-SP formulation, the highest weight loss was experienced, which can be due to the exiting of water from the co-polymer’s hydrophilic branch. As the temperature during the formulation process does not exceed the starting points of weight loss, it can be claimed in corroboration with the DSC results that MGA does not suffer from degradation during the process and that it is a safe method used to formulate these systems.

#### 2.2.2. X-ray Powder Diffraction Measurements

**Figure 4:**
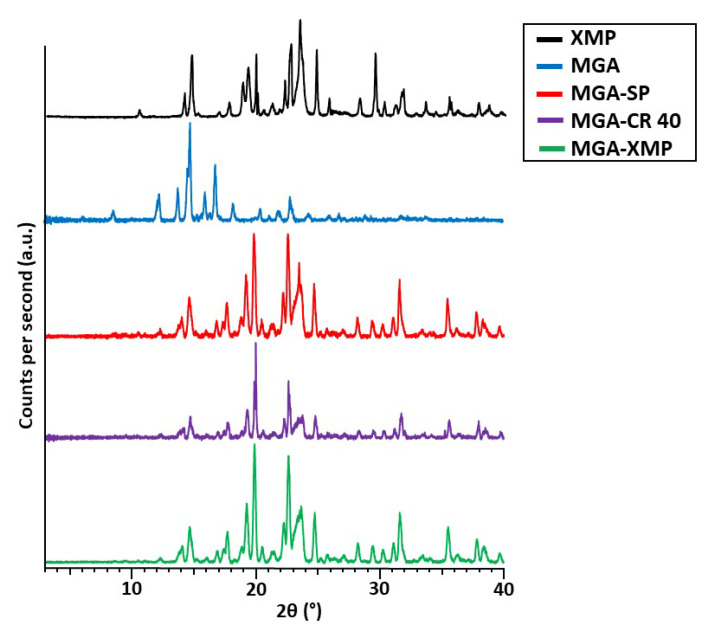
X-ray powder diffractograms of starting MGA, the carrier XMP, the surfactant-free MGA-XMP granule, and the surfactant-containing MGA-CR 40 and MGA-SP granules.

X-ray powder diffraction measurements were carried out to capture the diffractograms of each formulation and to determine their crystallinity state, as seen in Figure 4.

From the diffractograms, it can be claimed that the granules have a crystalline structure in all three cases, and the diffractogram of the carrier XMP corresponds to the characteristic crystalline peaks of the drug-containing formulations. Two characteristic peaks of MGA (13.51 and 16.02°) can be found with decreased peak intensity in the granule formulations, which assumes that some details of the MGA that were not able to be closed by the surfactants remained in the carrier in recrystallized form. The solubilizers are amorphous in nature; therefore, the peaks of the enclosed MGA can be hindered in this measurement as well.

#### 2.2.3. Granule Size and Distribution

The granule size and granule size distribution were measured via laser diffraction, the Span was calculated, and specific surface area (SSA) data were obtained from the measurement as seen in Table 4.

Based on the granule size measurements, MGA-SP showed the highest decrease in granule size because of its adequate consistency during granulation compared with the other compositions, while in the case of MGA-CR 40, a higher granule size was obtained due to the sticky nature of dispersed granule mass. There was no remarkable difference in the Span value of the starting MGA and MGA-containing formulations, which assume similar granule size distributions. The specific surface area was increased in the case of MGA-SP and MGA-CR 40 compared with MGA-XMP; therefore, it predicts higher dissolution in water alongside with a smaller granule size and the solubilizing capacity of these surfactant-type molecules.

#### 2.2.4. Flowability Studies

Important parameter of the granules is the flowability, which is closely related to the manufacturing-packaging processes in the industry. The flowability parameters of MGA-loaded granules were determined as seen in Table 5.

It can be concluded that the solubilizer containing samples have longer flow time as well as form higher angles of repose compared with the surfactant-free granule, especially in case of MGA-CR 40 because of its stickier consistency. In a comparison of the two solubilizers, CR 40 and SP, in the case of MGA-SP, improved flow properties can be experienced. The bulk density of the granules show that, by applying solubilizers, a more porous structure can be gained, which helps the infiltration of water when the product is dissolved.

### 2.3. Characterization of the Effect of Solubilizers

#### 2.3.1. Dynamic Light Scattering and Zeta Potential Measurements

Dynamic light scattering (DLS) measurements were carried out to characterize the average hydrodynamic diameter (expressed as Z-average), size distribution (polydispersity index—PdI), and the zeta potential was also measured (Table 6). The dissolved granules were filtered with a 0.45 µm polyether sulfone (PES) membrane to obtain this information and to filter out any excess carrier particle.

Based on the DLS results, it can be claimed that MGA-CR 40 has some disadvantages as these small particles may have a more beneficial surface-area-to-size ratio; in this size range, nanotoxicity may appear as a problem [15]. On the other hand, the MGA-SP formulation has the higher particle size, but it is in the corresponding range of polymeric micelles. The particles are in monodisperse distribution, under the PdI value of 0.500, meaning a uniform absorption profile could be achieved. The negative surface charges keep the colloidal solution stable as the particles are in favor of repelling each other and lower the tendency to aggregate. The absolute value for zeta potential is higher for the MGA-SP formulation, meaning higher repelling forces are presented, and it offers increased stability in aqueous solutions compared with CR 40.

#### 2.3.2. Surface Free Energy, Polarity, and Thermodynamic Solubility

The wettability of the granules has a significant influence on the dissolution rates and the release characteristics in oral drug delivery. Wettability is characterized by the contact angle of the liquid on the surface of the pressed granules, which in the case of this dosage form is the aqueous gastric acid. A smaller contact angle against water indicates greater wettability of the granule. To formulate an appropriately dissolving granule, a good wetting surface is a fundamental criterion. The polarity percentage can be calculated from the interfacial tension of the polar component (γ^p^), the interfacial tension of disperse component (γ^d^), and the surface free energy (γ). The results are presented in Table 7.

The results show that the carrier increases the surface free energies and the polarity in the case of the MGA-loaded formulations. MGA-CR 40 has worse wetting properties than MGA-SP, and its contact angles are higher than those of the solubilizer-free MGA-XMP formulation. This is due to the fact that CR 40 itself is a less polar surfactant compared with SP. However, it still increases the polarity of the MGA; therefore, it is suitable for administration. Since polarity affects the dissolution rate of a formula, these results predict that the MGA-SP formulation might have favorable drug-release kinetics as well. Besides polarity, the thermodynamic solubility was measured and solubility-related parameters were calculated to describe the effect of solubilizing agents on MGA (Table 8).

The results of the calculated parameters related to the thermodynamic solubility show that the poor water solubility of MGA (0.111 mg/mL) is increased in the presence of the surfactants. In the MGA-SP formulation, a higher increase can be experienced, which is supported by the higher molar solubilization capacity (χ) and surfactant–water partition coefficient (P). Based on the calculated standard free energy of solubilization, it can be claimed that dissolutions in the presences of CR 40 and SP are both thermodynamically favorable, but for MGA-SP, this value also has a larger absolute value.

#### 2.3.3. Solubilizing Efficiency

The solubilizing efficiency, as the percentage of MGA in dissolved state compared with the measured MGA, was determined by the filtration method. The excess suspended MGA was separated from the MGA micelles and dissoluble MGA, and the filtrate’s MGA concentration was determined by HPLC. The solubilizing efficiency percentages in the cases for MGA-CR 40 and MGA-SP were 37.4 ± 1.9 and 56.77 ± 2.6, respectively. SP is a polymeric micelle-forming agent with lower CMC value than CR 40 and is commonly applied to increase the solubility of poorly water-soluble drugs. The higher solubilizing efficiency result corroborated the increased water solubility, wettability, and polarity values. This also predicts a higher drug release in aqueous media, e.g., simulated gastric fluids.

### 2.4. In Vitro Gastric Drug Release Study

**Figure 5:**
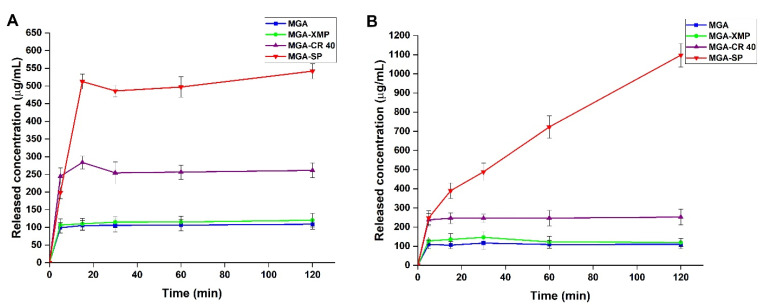
In vitro drug-release curves at simulated gastric conditions. (**A**) fasted state conditions, (**B**) fed state conditions. Data are presented as means ± SD (*n* = 3).

In vitro gastric drug release study was performed in two different media: Fasted State Simulated Gastric Fluid (FaSSGF) and Fed State Simulated Gastric Fluid (FeSSGF). The sink conditions were respected as calculated from the measured solubility of the granules and the raw MGA. The drug releases curves can be seen on Figure 5.

Based on the drug-release curves, in both media, significant increases were experienced in the case of the surfactant containing granules compared with the raw MGA and MGA-XMP (MGA-SP vs. MGA-XMP, MGA, ** *p* < 0.01; MGA-CR40 vs. MGA-XMP, MGA, * *p* < 0.05). Regarding the two surfactant-containing formulations, MGA-SP had an even more significant increase in the released drug effect in fasted conditions (MGA-SP vs. MGA-CR 40, * *p* < 0.05) and in fed conditions (MGA-SP vs. MGA-CR 40, ** *p* < 0.01). Based on the calculated difference factor (f_1_) and similarity factor (f_2_) of the drug-release curves of MGA (f_1_ = 4.43; f_2_ = 86.26), MGA-XMP (f_1_ = 9.19; f_2_ = 61.68) and MGA-CR 40 (f_1_ = 5.51; f_2_ = 64.72) are similar in both conditions; only a slightly higher concentration in the fed state study can be observed. In the fasted state, the burst effect can be observed in the 15 min measurement point for MGA-CR 40 and MGA-SP, which is a characteristic of the surfactant-mediated drug release with formulations in the nano-size range. Comparing the two curves of MGA-SP, in the fasted state, the equilibrium was almost set with a slight increase in the last measurement point; however, this cannot be observed in the fed state study. After two hours, the released concentration still increased with a high slope, which means that the most favorable drug release in the stomach can be achieved when the patient takes this formulation after meals.

Six different models were fitted to the drug release curves in both cases. In Table 9, the kinetic models and the calculated parameters can be found for the case of the fasted state study.

The drug release of MGA and MGA-XMP had the best fit (R^2^ 0.8689 and 0.9619) in the Korsmeyer–Peppas model, which means that the drug release is mainly influenced by the Fickian diffusion as their *n* values are lower than 0.05. The Higuchi kinetic best fit the surfactant-containing MGA-CR 40 and MGA-SP granules. It is corroborated the basic assumptions of the Higuchi model: the initial concentration of the MGA in the formulation is higher than the solubility in the XMP plus surfactant carrier; the MGA’s average hydrodynamic diameter is smaller than the size of the XMP carrier; the swelling of the system is insignificant; the drug diffusivity does not change; and the sink conditions are ensured. The drug-release model parameters were calculated for the fed state study as well, where the same conclusions can be claimed (Table 10). In the fed state study, the R^2^ values are relatively close to each other and does not show much difference between each other compared with the other fittings. Based on this, in the fed state, although Higuchi kinetic has the highest correlation, a mixed drug-release kinetic can be assumed.

## 3. Discussion

This study focused on the successful incorporation of MGA in the sugar alcohol-based carrier system with and without surfactants after the quality-by-design-based risk assessment process and on characterizing the structural and in vitro behavior associated with the change in water solubility of the water-redispersed granules based on the critically severe quality attributes and process parameters. MGA was successfully incorporated into the crystalline carrier system using melt technology as thermoanalytical and X-ray powder diffraction studies have demonstrated. Using surfactants, the melting enthalpy values were decreased, meaning that lower energy was required to formulate these granules and helped with the formulation process. MGA remained partially in a crystalline form in the granules; however, the amorphous surfactants increased the water solubility by physical encapsulation of MGA. The granule size of MGA-CR 40 was higher (D [0.5] = 408.951 ± 29.74 µm) compared with MGA-SP (D [0.5] = 223.822 ± 12.87 µm). This can be due to the stickier consistency of CR 40 compared with SP. Furthermore, the increase in the specific surface area (0.0247 and 0.0806 m^2^/g, for MGA-CR 40 and MGA-SP, respectively) in comparison with MGA-XMP (0.0153 m^2^/g) also helped with the granule dispersion in water; therefore, the main granule dosage form criteria were met. The flowability study revealed that MGA granules have a more porous structure in the case of MGA-SP formulation based on the bulk density (0.77 g/mL), which helps with the infiltration of water during the dispersion step.

The increase in water solubility was determined by characterizing the CR 40 micelles and SP polymeric micelles. The water solubility of MGA (0.111 mg/mL) was increased in both cases (0.739 and 2.154 mg/mL, respectively); however, in case of the MGA-SP formulation, higher water solubility, and favorable thermodynamic solubilizing energy and polarity (44.45%) were experienced. This result corroborated the higher encapsulation efficiency as well (37.4 and 56.77% for MGA-CR 40 and MGA-SP, respectively). The value-added properties of polymeric micelle-forming co-polymers can be justified with these results. The polymeric micelles are colloidally more stable than CR 40 micelles, indicated by the higher surface charge value (−12.99 mV), and most importantly, this results in stable particle size after administration. SP itself also has great circulation and dilution stability, as studies have proven before. MGA-SP had higher average hydrodynamic diameter values, but it is preferable due to nanotoxicological issues.

An in vitro gastric release study was performed to determine the drug-release kinetic of the granule formulations compared with raw MGA and the surfactant-free MGA-XMP formulation. The biorelevant approach was applied where the formulations were tested in fasted and fed state gastric conditions as well. In both cases, the MGA-SP formulation surpassed all other formulations, but most importantly, in the fed state, no equilibrium could have been experienced, indicating that the preferable administration of MGA-SP granule could be after meals. The Higuchi kinetic followed the CR 40 micelle and SP polymeric micelle drug-release kinetic; however, other strong correlations for other drug release models were found in the case of MGA-SP, indicating that a mixed drug-release kinetic is most plausible.

## 4. Materials and Methods

### 4.1. Materials

The carrier forming materials D-Xylitol (XYL), β-D-mannitol (MAN), and polyethylene glycol 6000 (PEG 6000) were purchased from Sigma-Aldrich (Budapest, Hungary). The solubilizing agents Cremophor^®^ RH 40 (CR 40) and Soluplus^®^ (SP) were obtained from BASF GmbH (Ludwigshafen, Germany). Megestrol-acetate (Farmabios S. p. A., Pavia, Italy) was chosen as the model material for this experimental work. Analytical grade solvent methanol was also purchased from Sigma-Aldrich. Powders for the biorelevant gastric fluids were purchased from Biorelevant.com Ltd. (London, UK). Distilled water was purified for the experiments using the Millipore Milli-Q^®^ (Merck Ltd., Budapest, Hungary) 140 Gradient Water Purification System.

### 4.2. QbD-Based Risk Assessment Process

At first, the Quality Target Product Profile (QTPP) was determined, followed by the selection of Critical Quality Attributes (CQAs)). A comparative analysis was performed, which proved to be useful in prior studies [16]. The interdependence rating was performed between the CR 40 micelles and SP polymeric micelles to the selected QTPP and CQA elements, respectively. A three-level scale (High-Medium-Low) was assigned to each relationship between the formulations and the QTPP/CQA elements. The three-level assigned represented the value of the influence of these factors to each other. The assessment was performed by the LeanQbD^®^ Software (QbDWorks, LLC, Austin, TX, USA). The final step was to quantify these risks based on probability rating, where the resulting severity scores were plotted as severity scores and the values were further compared [17,18,19].

### 4.3. Development of the Carrier Composition

For the preparation of the carrier, melt granulation was applied. XYL, MAN, and PEG 6000 were melted together on a sand bath at 112 °C. As the melt was cooled down at 33 °C, it was forced through a sieve with a mesh size of 1.2 mm to redisperse into granules. The process parameters and composition can be seen on Table 11.

### 4.4. Incorporation of Solubilizers and MGA in the Carrier

After the development of the carrier composition, the two solubilizing agents and MGA were incorporated in the carrier. Three different batch of XYL, MAN, and PEG 6000 were melted together on a sand bath at 112 °C. In the first batch, 1.6991 g of CR 40 and, in the second, 1.60 g of SP were dispersed; the third remained solubilizer free. Each sample was cooled down at 80 °C, and 0.40 g of MGA was dispersed in them. The amounts are for a single dose of 400 mg of MGA. Before the total solidification of the melt at 33 °C, the mass was dispersed through a sieve with a mesh size of 1.2 mm, forming solid granules.

### 4.5. Characterization of the MGA Containing Carrier in Solid State

#### 4.5.1. Differential Scanning Calorimetry

DSC measurements were carried out to characterize the thermal behavior of MGA-containing granules with and without the surfactants. Data were recorded with a Mettler-Toledo 821e DSC instrument (Mettler-Toledo GmbH, Gießen, Switzerland). Samples of 5 ± 0.2 mg were placed in aluminum pans and were examined in the temperature interval of 25–240 °C at a heating rate of 10 °C/min under a constant argon flow of 100 mL/min. Data were analyzed by STARe software (version 16.0). Each measurement was normalized to the sample size.

#### 4.5.2. Thermogravimetry

TGA measurements were carried out to investigate the thermal stability of the carrier composition with and without the surfactants. Data were recorded with a Mettler-Toledo TGA/DSC 1 (Mettler-Toledo GmbH, Gießen, Germany) instrument; 5 ± 0.2 mg of the formulations was measured into aluminum pans, closed, and inserted into the furnace. The furnace was heated up from 25 to 240 °C with a 10 °C/min heating rate, and the weight change was recorded in accordance with the temperature increase. The data were analyzed by STARe software.

#### 4.5.3. X-ray Powder Diffraction

XRPD analysis was performed using a Bruker D8 Advance diffractometer (Bruker AXS GmbH, Karlsruhe, Germany) with Cu K λI radiation (λ = 1.5406 Å) and a VÅNTEC-1 detector. Samples were scanned at 40 kV and 40 mA. The angular range was 3 to 40° 2θ, at a step time of 0.1 s and a step size of 0.007°. The sample was placed on a quartz holder and measured at ambient temperature and ambient relative humidity. All manipulations, including Kα2-stripping, background removal, and smoothing of the area under the peaks of the diffractograms were performed using the DIFFRACTPLUS EVA software (version 13.0.0.1).

#### 4.5.4. Determination of Granule Size and Distribution

Laser diffraction method was used to determine the granule size and granule size distribution of the formulations compared with the initial MGA (Malvern Mastersizer Scirocco 2000, Malvern Instruments Ltd., Worcestershire, UK). The refractive index of MGA (1.551) was set. The dry dispersion unit was used for the measurements. Approximately 1.0 to 2.0 g of the samples was loaded into the feeding tray. The dispersion air pressure was adjusted to 3 bar, and 75% vibration feed was used. The measurements were carried out in triplicate. The granule size distribution was characterized by the following parameters: D [0.1] (10% of the volume distribution is below this size), D [0.5] (the volume median diameter), and D [0.9] (90% of the volume distribution is below this value). Specific surface area (SSA) and the Span value were also obtained from the measurements.

#### 4.5.5. Flowability Studies

The parameters of flowability were determined with software-controlled PharmaTest PTG-1 powder testing equipment (PharmaTest, Hainburg, Germany). The powder flow was controlled by sensors, and the time was measured for 100 mL of spray-dried material to flow; the angle of repose (α), the volume of the heap, and the bulk density of the sample are calculated from the mass and height of the formed powder heap.

### 4.6. Characterization of the Effect of Solubilizers

#### 4.6.1. Dynamic Light Scattering and Zeta Potential Measurements

The average hydrodynamic diameter (Z-average), polydispersity index (PdI), and the zeta potential were measured using a Malvern Zetasizer Nano ZS (Malvern Instruments, Worcestershire, UK). The solubilizer-containing carrier formulations were dispersed in purified water and then measured at 25 °C in folded capillary index. The refractive index was 1.551. The measurements were carried out in triplicate with independent formulations.

#### 4.6.2. Surface Free Energy and Polarity Investigations

The melted compositions were cast on a slide, where they solidified with a flat surface on which contact angle measurements were carried out. The contact angle (θ) was determined by means of the sessile drop technique, using an OCA 20 Optical Contact Angle Measuring System (Dataphysics, Filderstadt, Germany). The wetting angles of the casts were determined after 4.3 µL of purified water had been dropped onto the surface of the castings. The change in the wetting angle was registered from 1 to 30 s using a circle fitting method of the OCA System. The method of Wu was applied, in which two liquids with known polar (γ_i_^p^) and dispersion (γ_i_^d^) components are used for the measurement. The solid surface free energy is the sum of the polar (γ_i_^p^) and non-polar (γ_i_^d^) components and was calculated according to the Wu equation [20]:

(1) $$\left(1+cos\Theta \right)\gamma_{l}=\frac{4\left(\gamma_{s}^{d}\gamma_{l}^{d}\right)}{\gamma_{s}^{d}\gamma_{l}^{d}}+\frac{4\left(\gamma_{s}^{p}\gamma_{l}^{p}\right)}{\gamma_{s}^{p}\gamma_{l}^{p}}$$

where *Θ* is the contact angle, *γ_s_* is the solid surface free energy, and *γ_l_* is the liquid surface tension. The percentage polarity can be calculated from the *γ^p^* and *γ* values: (*γ^p^*/*γ*) × 100. The solvents were purified water (*γ^p^* = 50.2 mN/m, *γ^d^* = 22.6 mN/m) and diiodomethane (*γ^p^* = 1.8 mN/m, *γ^d^* = 49 mN/m).

#### 4.6.3. Thermodynamic Solubility

The thermodynamic solubility of MGA and MGA-containing formulations were determined in purified water (pH = 7.02; κ = 0.05 µS/cm) at 25 °C. Each formulation was slowly dispersed in 1 mL of water until visible oversaturation. The mixtures were stirred constantly with a magnetic stirrer for 72 h. After that, they were filtered through a 0.45 µm polyether sulfone (PES) membrane and the content of the dissolved drug was determined with HPLC [21].

Using the date received from the solubility test, the parameters related to the solubility enhancement effect of surfactants were calculated [22].

1. Molar solubilization capacity (*χ*) or moles of drug that can be solubilized per mol of surfactants

(2) $$\chi =\frac{S_{tot}-S_{w}}{C_{surf}-CMC}$$

2. Surfactant–water partition coefficient (*P*), which is the ratio of the drug concentration solubilized by the surfactant to the drug concentration in water

(3) $$P=\frac{S_{tot}-S_{w}}{S_{w}}$$

3. Standard free energy of solubilization (Δ*G_s_*^0^), estimated from the molar surfactant-water partition coefficient (*P_M_*)

(4) $$\Delta G_{s}^{0}=-RT\cdot ln\frac{\chi \cdot \left(1-CMC\right)}{S_{w}}=-RT\cdot ln\left(P_{M}\right)$$

In the equations, *S_tot_* means the total solubility of MGA with the presence of surfactants, *S_w_* is the solubility of MGA in water, *CMC* is the critical micelle concentration, C_copol_ is the copolymer concentration in each solution, *R* is the universal constant of gases, and *T* is temperature.

#### 4.6.4. Solubilizing Efficiency

To determine the solubilized drug amount, formulations were dispersed in 5 mL of purified water; then, 1 mL of the dispersed solution was transferred into a Spin-X^®^ centrifuge tube filter. It has a cellulose acetate membrane filter with a 0.22 µm cut-off pore diameter, placed in a 2 mL polypropylene tube (Costar, Salt Lake City, UT, USA). Then, the unentrapped MGA was separated using a Hermle Z323K high performance refrigerated centrifuge (Hermle AG, Gossheim, Germany) at 17,500 rpm and 25 °C for 45 min. The MGA amount remaining on the filter was dissolved in 3 mL of methanol and was quantified by HPLC. By this method, the insolubilized drug amount can be determined, and the remaining amount is divided into the dissolved MGA and the encapsulated MGA in the ratio is calculated from the surfactant–water partition coefficient (P) [21].

### 4.7. In Vitro Gastric Drug-Release Study

The modified paddle method (Hanson SR8 Plus (Teledyne Hanson Research, Chatsworth, CA, USA) was used to examine the rates of drug release for the surfactant-free and surfactant-containing MGA granules; 5 g of granules were dissolved in 10 mL of water, and then, they were placed in dialysis membranes (Spectra/Por^®^ Dialysis Membrane with a MWCO value of 12–14 kD (Spectrum Laboratories Inc., Rancho Dominguez, CA, USA). The drug-release study was performed in 200 mL of dissolution media at 37 °C. The paddle was rotated at 100 rpm, and the sampling was performed for up to 120 min in predefined intervals. Three parallel measurements took place for the granules. Quantification of aliquots was performed by HPLC after filtration with a PES membrane (0.45 µm). To present the biorelevant drug-release data, two separate dissolution media were used: Fasted State Simulated Gastric Fluid (FaSSGF) and Fed-State Simulated Gastric Fluid (FeSSGF) [23,24]. The difference factor (*f_1_*) and similarity factor (*f_2_*) were calculated to compare the dissolution profile of initial MGA and MGA-containing formulations [25]. Difference factor (*f_1_*) is the percentage difference between two curves at each point and is a measurement of the relative error between the two curves, calculated as follows:

(5) $$f_{1}=\left\{\frac{\sum_{t=1}^{n}\left|R_{t}-T_{t}\right|}{\sum_{t=1}^{n}R_{t}}\right\}\times 100$$

The similarity factor (*f_2_*) is a logarithmic reciprocal square root transformation of the sum of squared error and is a measurement of the similarity in the percent (%) dissolution between the two curves:

(6) $$f_{2}=50\;log\left\{{\left(1+\frac{1}{n}\overset{n}{\underset{t=1}{\sum }}{\left(R_{t}-T_{t}\right)}^{2}\right)}^{-0.5}\times 100\right\}$$

where *n* is a number of time points, and *Rt* and *Tt* are the mean percentages of the released drug from the (*R*) and (*T*) products, respectively, at the time point *t*, 1 ≤ *t* ≤ *n.* Two dissolution profiles are considered similar and bioequivalent if f_1_ is between 0 and 15 and if *f_2_* is between 50 and 100 [25].

### 4.8. Modelling the In Vitro Drug-Release Kinetics

The release kinetics of MGA from the granule formulations was compared with the drug-release kinetics of the initial MGA. Six mathematical drug-release models (zero order, first order, second order, Hixson–Crowell, Higuchi, and Korsmeyer–Peppas models) were fitted with the obtained cumulative drug release vs. time curves to describe the kinetics. To evaluate which model had the best fit, the values of regression coefficient (R^2^) were determined and compared [26,27].

### 4.9. Quantitative Analysis of MGA via HPLC

For determining the concentration during our investigations, a high-performance liquid chromatography (HPLC) analysis was performed using an Agilent 1260 Infinity (Agilent Technologies, Santa Clara, CA, USA). As stationary phase, a Luna^®^ C8(2) liquid chromatography column (5 µm, 250 × 4.6 mm, 100 Å (Phenomenex, Torrance, CA, USA)) was used; 10 µL of the samples were injected to determine the concentration of MGA. The temperature was set at 50 °C. The mobile phases used were purified water (A) and methanol (B). The separation was performed in three steps by gradient elution. The proportion of starting 25% A eluent was reduced to 10% in 8 min; then, it remained at this ratio for 12 min and then was raised again to 25% are 14 min. The eluent flow rate was set at 0.5 mL/min, and the chromatograms were detected at 254 ± 4 nm using a UV–Vis diode array detector. Data were evaluated using ChemStation B.04.03. Software (Agilent Technologies, Santa Clara, CA, USA). The retention time of MGA was 11.27 min. The linear regression of the calibration line was 0.999. The determined limit of detection (LOD) and quantification (LOQ) in the case of MGA were 2.573 and 7.797 ppm, respectively.

## 5. Conclusions

Quality-by-design-driven melt technology was proposed for the development of MGA-containing granules, where the use of a polymeric micelle former (SP) in the crystalline carrier system (XMP) was more efficient in terms of solubility enhancement and drug release compared with the classic micelle former (CR 40).

From a practical point of view, the developed granule can be dosed and filled into a sachet according to the desired dose, which can be easily redispersed in water before administration. In the applied concentration of both surfactants, no adverse reactions occurred. Moreover, its sweet taste due to the sugar alcohols (XYL and MAN) applied can further support patient compliance.

## Figures and Tables

**Table 1:** Comparison of technical properties of Cremophor^®^ RH 40 and Soluplus^®^.

Property	Cremophor^®^ RH40 (CR 40)	Soluplus^®^ (SP)
Molecular weight (M_r_)	853.91	90,000–140,000
Critical micelle concentration (CMC)	9 mg/mL	7.6 mg/mL
Hydrophilic–lipophilic balance (HLB)	14–16	13
Oral median lethal dose in rat (LD_50_)	>10,000 mg/kg	>5000 mg/kg

**Table 2:** Melting points (T_m_), as the peaks of the endothermic curves; the onset–endset temperatures of these peaks; and the melting enthalpy (ΔH_m_) of the characteristic melting points of MGA and the carrier system in the granule formulations.

	T_Onset_ (°C)	T_m_ (°C)	T_Endset_ (°C)	ΔH_m_ (J/g)
**MGA**				
	217.47	217.95	219.68	−59.76
**XMP**				
PEG 6000	58.36	60.45	62.20	−42.06
XYL-MAN	89.25	91.24	93.14	−115.78
**MGA-XMP**				
PEG 6000	58.60	60.42	62.25	−42.15
XYL-MAN	89.23	91.28	93.28	−116.83
**MGA-CR 40**				
PEG 6000	56.49	59.06	60.66	−37.06
XYL-MAN	88.94	91.19	93.18	−90.02
**MGA-SP**				
PEG 6000	57.81	60.02	62.32	−32.61
XYL-MAN	89.27	91.21	93.18	−86.18

**Table 3:** Thermal degradation properties of MGA and the MGA-containing granule formulations.

Material	Starting Point of Weight Loss (°C)	Maximal Weight Loss at 240 °C (%)
MGA	232	0.49
MGA-XMP	234	2.15
MGA-CR 40	227	2.75
MGA-SP	225	2.79

**Table 4:** Granule size distribution and SSA obtained by laser diffraction.

Sample	D [0.1] (µm)	D [0.5] (µm)	D [0.9] (µm)	Span	SSA (m^2^/g)
MGA	1.154 ± 0.06	2.957 ± 0.19	6.215 ± 0.13	1.712	2.34
MGA-XMP	169.389 ± 11.45	844.079 ± 31.54	1531.465 ± 37.33	1.614	0.0153
MGA-CR 40	112.32 ± 7.49	408.951 ± 29.74	797.749 ± 64.19	1.802	0.0247
MGA-SP	20.844 ± 2.12	223.828 ± 12.87	387.744 ± 8.75	1.639	0.0806

**Table 5:** Flow time, angle of repose, and the bulk density of the samples calculated from the mass and height of the formed powder heap. Data are presented as means ± SD (*n* = 3).

Sample	Flow Time (s)	Angle of Repose (°)	Bulk Density (g/mL)
MGA-XMP	10.6 ± 0.2	24.33 ± 0.12	1.00
MGA-CR 40	24.1 ± 0.5	34.77 ± 1.05	0.88
MGA-SP	18.5 ± 0.4	29.16 ± 0.78	0.77

**Table 6:** Average hydrodynamic diameter (Z-average), polydispersity index (PdI), and zeta potential measurement results of MGA-CR 40 and MGA-SP formulations. Data are presented as means ± SD (*n* = 3).

Sample	Z-Average (nm)	PdI	Zeta Potential (mV)
MGA-CR 40	27.82 ± 0.87	0.205 ± 0.010	−1.15 ± 0.55 mV
MGA-SP	102.27 ± 2.06	0.259 ± 0.006	−12.99 ± 0.11

**Table 7:** Contact angles and the related wetting parameters of MGA and MGA-containing granule formulations. Data are presented as means ± SD (*n* = 3).

Samples	Θ_water_ [°]	Θ_diiodomethane_ [°]	γ^d^ [mN m^−1^]	γ^p^ [mN m^−1^]	γ [mN m^−1^]	Polarity (%)
MGA	54.2 ± 4.3	4.7 ± 0.1	45.96	18.82	64.78	29.05
MGA-XMP	10.6 ± 0.2	7.4 ± 0.3	45.46	36.08	81.54	44.25
MGA-CR 40	30.4 ± 3.7	7.9 ± 2.1	45.46	30.03	75.49	39.78
MGA-SP	10.3 ± 2.2	9.2 ± 1.7	45.24	36.20	81.44	44.45

**Table 8:** Solubility of MGA (S_w_), the granules (S_tot_) with the calculated solubility related parameters: molar solubilization capacity (χ), surfactant–water partition coefficient (P), and standard free energy of solubilization (ΔG_s_^0^). Where data are not interpretable, a hyphen (-) is marked. Data are presented as means ± SD (*n* = 3).

Samples	S_w_ (mg/mL)	S_tot_ (mg/mL)	χ	P	ΔG_s_^0^ (kJ/mol)
MGA	0.111 ± 0.003	-	-	-	-
MGA-XMP	-	0.113 ± 0.006	-	-	-
MGA-CR 40	-	0.739 ± 0.010	0.00332	5.627	−8.724
MGA-SP	-	2.154 ± 0.009	0.01116	18.311	−11.857

**Table 9:** Kinetic models and the calculated parameters for the drug-release test performed in the Fasted State Simulated Gastric Fluid (FaSSGF).

Model		MGA	MGA-XMP	MGA-CR 40	MGA-SP
Zero order	k_0_ (µg min^−1^)	0.0645	0.0705	0.1563	0.3084
	R^2^	0.5768	0.5768	0.5518	0.675
	t_0.5_ (min)	775.19	709.22	319.89	162.13
First order	k_1_ (min^−1^) × 10^−3^	0.23	0.31	0.54	1.92
	R^2^	0.2194	0.2466	0.1688	0.427
	t_0.5_ (min)	3465.73	2310.49	1386.29	364.81
Second order	k_2_ (µg^−1^ min^−1^) × 10^−5^	0.21	0.36	0.69	2.21
	R^2^	0.2207	0.249	0.1681	0.4410
	t_0.5_ (min)	4814	3200.66	1494.5	381.55
Korsmeyer–Peppas	k_K-P_ (min^−*n*^)	20.83	19.87	7.89	11.89
	*n*	0.027	0.0361	0.0074	0.2768
	R^2^	0.8689	0.9619	0.028	0.6474
	t_0.5_ (min)	1203.14	1255.71	225.18	179.11
Higuchi	k_H_ (µg min^−1/2^)	0.7012	0.7623	1.7144	3.2341
	R^2^	0.8188	0.8281	0.7978	0.8918
	t_0.5_ (min)	5084.59	4302.17	850.58	239.02
Hixson–Crowell	k_H-C_ (µg^1/3^ min^−1^) × 10^−3^	1.12	1.17	2.53	5.37
	R^2^	0.5773	0.5901	0.5516	0.6801
	t_0.5_ (min)	957.56	870.51	383.02	180.67
Best fit		Korsmeyer–Peppas	Korsmeyer–Peppas	Higuchi	Higuchi

**Table 10:** Kinetic models and the calculated parameters for the drug-release test performed in the Fed State Simulated Gastric Fluid (FeSSGF).

Model		MGA	MGA-XMP	MGA-CR 40	MGA-SP
Zero order	k_0_ (µg min^−1^)	0.0708	0.0901	0.1504	0.5138
	R^2^	0.5815	0.6226	0.5546	0.9283
	t_0.5_ (min)	706.21	554.94	322.45	97.31
First order	k_1_ (min^−1^) × 10^−3^	0.37	0.49	0.51	6.13
	R^2^	0.2295	0.3241	0.1747	0.9759
	t_0.5_ (min)	2310.49	1732.87	1386.29	113.63
Second order	k_2_ (µg^−1^ min^−1^) × 10^−5^	0.32	0.48	0.51	95.1
	R^2^	0.2314	0.3305	0.2036	0.9888
	t_0.5_ (min)	3199.67	2380.25	1803.4	100.07
Korsmeyer–Peppas	k_K-P_ (min^-*n*^)	19.33	19.37	8.22	17.79
	*n*	0.0314	0.0615	0.0076	0.4604
	R^2^	0.9523	0.8944	0.0499	0.9861
	t_0.5_ (min)	1398.13	1077.51	895.41	94.30
Higuchi	k_H_ (µg min^−1/2^)	0.769	0.9613	1.6479	4.8605
	R^2^	0.823	0.8515	0.8001	0.9977
	t_0.5_ (min)	4227.53	2705.34	920.62	105.82
Hixson–Crowell	k_H-C_ (µg^1/3^ min^−1^) × 10^−3^	1.17	1.46	2.14	9.71
	R^2^	0.5810	0.6244	0.5546	0.9577
	t_0.5_ (min)	870.5	683.97	389.98	98.72
Best fit		Korsmeyer–Peppas	Korsmeyer–Peppas	Higuchi	Higuchi

**Table 11:** Components, composition, and main temperature conditions of the carrier system (XMP) development.

Component	Composition for Single Dose (g (% *w*/*w*))	Melt Forming Temperature (°C)	Solidifying Temperature (°C)
XYL	3.6875 (57.65)	112	33
MAN	0.9220 (14.41)		
PEG 6000	1.7869 (27.94)		

## Data Availability

The data presented in this study are available from the corresponding author upon request.
